# The dual transcriptional regulator CysR in *Corynebacterium glutamicum *ATCC 13032 controls a subset of genes of the McbR regulon in response to the availability of sulphide acceptor molecules

**DOI:** 10.1186/1471-2164-9-483

**Published:** 2008-10-14

**Authors:** Christian Rückert, Johanna Milse, Andreas Albersmeier, Daniel J Koch, Alfred Pühler, Jörn Kalinowski

**Affiliations:** 1 Institut für Systembiologie & Genomforschung, Universität Bielefeld, Universitätsstr. 25, D-33615 Bielefeld, Germany; 2 Division of Chemistry and Chemical Engineering, California Institute of Technology, 1200 E California Blvd, Pasadena, CA91125, USA; 3 Lehrstuhl für Genetik, Universität Bielefeld, Universitätsstr. 25, D-33615 Bielefeld, Germany

## Abstract

**Background:**

Regulation of sulphur metabolism in *Corynebacterium glutamicum *ATCC 13032 has been studied intensively in the last few years, due to its industrial as well as scientific importance. Previously, the gene *cg0156 *was shown to belong to the regulon of McbR, a global transcriptional repressor of sulphur metabolism in *C. glutamicum*. This gene encodes a putative ROK-type regulator, a paralogue of the activator of sulphonate utilisation, SsuR. Therefore, it is an interesting candidate for study to further the understanding of the regulation of sulphur metabolism in *C. glutamicum*.

**Results:**

Deletion of *cg0156*, now designated *cysR*, results in the inability of the mutant to utilise sulphate and aliphatic sulphonates. DNA microarray hybridisations revealed 49 genes with significantly increased and 48 with decreased transcript levels in presence of the native CysR compared to a *cysR *deletion mutant. Among the genes positively controlled by CysR were the gene cluster involved in sulphate reduction, *fpr2 cysIXHDNYZ*, and *ssuR*. Gel retardation experiments demonstrated that binding of CysR to DNA depends *in vitro *on the presence of either *O*-acetyl-L-serine or *O*-acetyl-L-homoserine. Mapping of the transcription start points of five transcription units helped to identify a 10 bp inverted repeat as the possible CysR binding site. Subsequent *in vivo *tests proved this motif to be necessary for CysR-dependent transcriptional regulation.

**Conclusion:**

CysR acts as the functional analogue of the unrelated LysR-type regulator CysB from *Escherichia coli*, controlling sulphide production in response to acceptor availability. In both bacteria, gene duplication events seem to have taken place which resulted in the evolution of dedicated regulators for the control of sulphonate utilisation. The striking convergent evolution of network topology indicates the strong selective pressure to control the metabolism of the essential but often toxic sulphur-containing (bio-)molecules.

## 1 Background

*Corynebacterium glutamicum*, a gram-positive soil bacterium, is of great biotechnological interest due to its ability to produce high yields of L-glutamate and L-lysine [1]. The industrial interest as well as the emergence of *C. glutamicum *as a model organism for the order of the *Actinomycetales *in general and specifically the suborder *Corynebacterineae *resulted in intensified research on the metabolic capabilities of *C. glutamicum*. One focus of this research has been the elucidation of the pathways involved in the metabolism of sulphur-containing amino acids (reviewed in [2]). This is in part due to the ability of *C. glutamicum *to produce high yields of L-lysine, which shares the precursor L-aspartic acid with L-methionine, and L-serine [3,4], the precursor for L-cysteine biosynthesis. From the existence of these strains it can be concluded that *C. glutamicum *has the capability to produce significant amounts of sulphur-containing amino acids, a hypothesis that is backed at least for L-methionine by *in silico *studies [5]. Yet, although almost all of the genes involved in the various pathways have been identified in the last few years [2], no production strain is available up to now.

This might be at least in part due to a tight transcriptional regulation of the genes involved in sulphur metabolism. The regulation has therefore been studied intensively in recent years, resulting in the identification of the global transcriptional repressor McbR (methionine and cysteine biosynthesis regulator) [6,7] which was shown to control almost all genes known to be involved in the various pathways of sulphur interconversion. Activity of McbR was shown to be negatively controlled by *S*-adenosyl-L-homocysteine (SAH) [7] which is derived from the methylation agent *S*-adenosyl-L-methionine (SAM). SAM is of great importance during cell growth as it is needed, e.g., for the modification of newly synthesised DNA [8], linking sulphur metabolism to the growth phase [7].

Still, studies from other model organisms like *Escherichia coli *or *Bacillus subtilis *implicated the presence of additional regulatory mechanisms to adapt to changing environmental conditions. For example, sulphur metabolism in *B. subtilis *is controlled globally on the RNA level via the S-box regulon in response to SAM availability [9], analogous to McbR control in *C. glutamicum*. In addition to this global regulation, a number of regulatory proteins involved in the control of sulphur metabolism have been described recently for *B. subtilis*, e.g. CysL [10], Spx [11], and CymR [12,13]. In *C. glutamicum*, targets for additional regulation were delivered by the McbR regulon itself. McbR controls two genes that are predicted to encode regulators of the ROK family [14]. One, now called SsuR (sulphonate sulphur utilisation regulator), was shown by Koch *et al*. [15] to control a subset of McbR-regulated genes involved in the utilisation of aliphatic sulphonates [16]. Thus, the other, encoded by *cg0156*, was likely to be involved in transcriptional regulation of sulphur metabolism in *C. glutamicum *also.

Therefore, the gene *cg0156 *and its encoded protein were analysed by means of growth assays of a defined deletion mutant strain, transcriptional studies, and electrophoretic mobility shift assays (EMSAs) to identify the regulon, binding sites, and effectors of this predicted transcriptional regulator.

## 2 Results

### 2.1 The *Corynebacterium glutamicum *mutant CR030 with a deleted *cg0156 *(*cysR*) gene can no longer grow with sulphate or sulphonates as sole source of sulphur

The transcriptional regulator McbR, discovered by Rey *et al*. [6], was shown to act as the global repressor of sulphur metabolism in *Corynebacterium glutamicum *ATCC 13032 [7]. Besides a large number of genes encoding enzymes, transporters, and unknown functions, the regulon of McbR includes two genes coding for possible regulators, *cg0012 *(*ssuR*) and *cg0156*. According to a bioinformatic classification, both encoded proteins belong to the ROK protein family [14], which is made up of sugar kinases, transcriptional repressors for sugar catabolism operons, and proteins of unknown function [17].

As a first step, a detailed bioinformatic analysis of the protein encoded by *cg0156 *was carried out. Cg0156 has a length of 381 amino acids and a molecular mass of 40.1 kDa. According to a similarity search against the SUPERFAMILY database [18], Cg0156 contains a helix-turn-helix (HTH) motif of the winged-helix type (amino acids 34–65) and shows weak similarities to members of the ROK protein family, a finding that is supported by a weak hit against the PFAM database [19] as well as against the CDD database [20]. Despite being somewhat similar to NagC [21] and Mlc (originally discovered as DgsA [22]) from *Escherichia coli*, the function of Cg0156 could not be inferred based on sequence similarity alone. A phylogenetic tree build from proteins with at least 20% similarity retrieved from THESEED [23] also revealed little information other than the existence of potential orthologues in *Corynebacterium efficiens *and *Corynebacterium diphtheriae*, while SsuR in *C. glutamicum *is most likely a paralogue (data not shown). This relationship as well as being a part of the McbR regulon indicated that Cg0156 might be a transcriptional regulator, involved in the regulation of sulphur metabolism in *C. glutamicum*.

**Figure 1:**
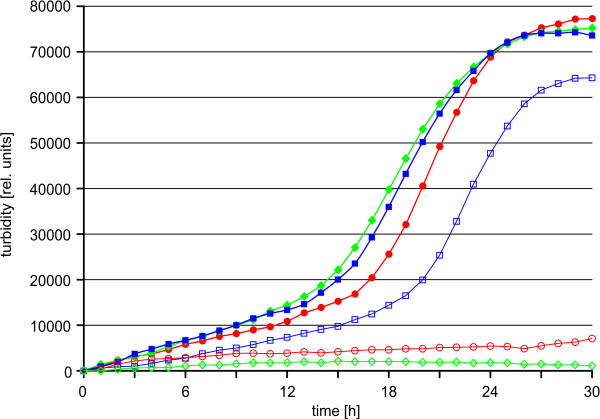
**Growth of the *C. glutamicum *wild type strain and the mutant strain CR030 (Δ*cysR*)**. The strains were cultured in liquid minimal medium containing the following sulphur source in 100 *μ*M concentration: sulphate (◆), L-cysteine (■) and ethanesulphonate (●). Empty symbols represent the growth of *C. glutamicum *CR030. The growth, monitored with a nephelometer, is shown as relative turbidity, displaying the mean of three biological replicates with six technical replicates each.

To obtain a hint on the function of Cg0156, a defined deletion mutant (CR030) was constructed, almost completely removing the open reading frame. Like the assay performed by Koch *et al*. [15] to characterise SsuR, the growth of CR030 was then assayed in liquid MMES minimal medium containing different compounds as sole source of sulphur, by name sulphate, ethanesulphonate, and L-cysteine, and compared to the wild type strain ATCC 13032 (Fig. 1). While the wild type strain grows equally well with all three compounds tested, the mutant strain CR030 can grow with neither sulphate nor ethanesulphonate, while growth on L-cysteine is comparable to that of the wild type albeit with a slight increase in duplication time and a slightly decreased final biomass. In addition, neither sulphite nor any of the aliphatic sulphonates (as well as their esters) that can be utilised by the wild type [16] can be used by the mutant (data not shown). Based on the apparent requirement for cysteine, Cg0156 was designated CysR (**cys**teine biosynthesis **r**egulator). This raised the question of which genes are the target(s) of CysR-mediated transcriptional control. The most likely candidates were the genes involved in the pathway for assimilatory reduction of sulphate, *fpr2 cysIXHDNYZ *(as proposed earlier by Rückert *et al*. [24]), the genes needed for L-cysteine biosynthesis (*cysKE*), and the genes involved in sulphonate(ester) utilisation (*ssu *and *seu *[16]).

### 2.2 The regulator CysR affects a subset of genes of the McbR regulon

The first step to determine the targets of CysR was a global transcriptome study using the whole *C. glutamicum *genome microarray developed by Hüser *et al*. [25]. Due to the observation that the mutant CR030 looses the ability to grow on sulphate and sulphonates, it was reasoned that CysR might act as an activator of the genes involved. Therefore, the mutant as well as the wild type strain were grown in MMS minimal medium with limiting amounts of L-cysteine (to allow for growth of CR030) as sole source of sulphur and were then subjected to sulphur starvation for 30 min to maximise transcription of the *ssu*, *seu*, and *cys *genes (as observed by Koch *et al*. [16] and Rückert *et al*. [24]). Isolation of total RNA and DNA microarray hybridisations were performed, revealing 82 genes with reduced mRNA levels in the mutant and 20 genes with increased mRNA levels (data not shown). As expected, all genes of the *fpr2 cysIXHDNYZ *cluster as well as all *ssu *and *seu *genes, including *ssuR*, were found among the genes with strongly reduced transcription. These results indicated a function as transcriptional activator for these genes but raised the question whether CysR affects the transcription of the *ssu *and *seu *genes directly or indirectly via transcriptional regulation by SsuR. Furthermore, an additional 16 genes of the McbR regulon were found less transcribed in the mutant, e.g. *cysK*, *cg3372*, and *cg2678-74*.

**Figure 2:**
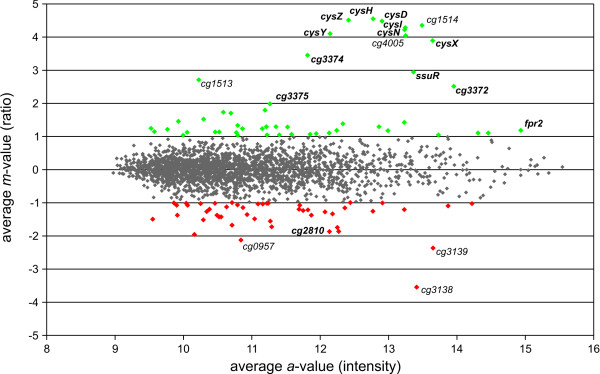
**Ratio/intensity plot deduced from DNA microarrayexperiments comparing the transcriptome of *C. glutamicum*CR031 (*mcbR*^ΔHTH ^*ssuR*^ΔHTH ^*cysR*^const^) with that of *C. glutamicum *CR032 (*mcbR*^ΔHTH ^*ssuR*^ΔHTH^*cysR*^ΔHTH^)**. The analysed strains were grown in minimal medium with 2 mM L-cysteine as sole sulphur source to o.D.600 10 and then subjected to sulphur starvation for 30 min. Genes showing a strong (|m| ≥ 2) differential expression are named and displayed as green respectively red spots, those without significant differential expression are given as grey spots. Genes belonging to the McbR regulon are marked in bold type.

To differentiate between direct effects of CysR and indirect effects due to, e.g., regulation of McbR and SsuR, two new mutants were constructed two minimise these indirect effects: CR031 (*mcbR*^ΔHTH ^*ssuR*^ΔHTH ^*cysR*^const^), a mutant constitutively expressing CysR while lacking active forms of the McbR and SsuR proteins, and CR032 (*mcbR*^ΔHTH ^*ssuR*^ΔHTH ^*cysR*^ΔHTH^) which expresses all three regulators in inactive forms. By removing activity of the two known regulators *mcbR *and *ssuR *in both mutants, only CysR-dependent regulation should remain. Deleting only the helix-turn-helix (HTH) DNA-binding domains of the regulators instead of full-length deletion allowed for quantification of the remaining transcript. Finally, replacing the native promoter of *cysR *by the constitutive promoter of the *neo *gene [26], a possible transcriptional auto-regulation of *cysR *is prevented in the mutant strain CR031. As a prerequisite, the native promoter of *cysR *was identified by mapping the transcription start site using the 5'-RACE method (data not shown). It was found that the transcriptional start site is identical to the start site of translation, adding *cysR *to the growing list of *C. glutamicum *genes transcribed as leaderless transcripts. Both strains were grown in MMS minimal medium with 2 mM L-cysteine as sole source of sulphur to o.D._600 _10 and then subjected to sulphur starvation as done previously. Applying microarray analysis, 49 genes were found to have increased mRNA pools in the mutant strain CR031 constitutively expressing an intact CysR, compared to the HTH deletion mutant CR032, while 48 genes had reduced mRNA levels (Fig. 2). Among those genes with the highest increase in transcript abundance were again several genes that also belong to the McbR regulon, by name *cysIXHDNYZ*, *fpr2*, *ssuR*, and *cg3372-75*, supporting the notion that CysR might act as a transcriptional activator on these genes. Interestingly, neither the SsuR-controlled *ssu*/*seu *genes nor the rest of the McbR-regulated genes detected in the comparison of the wild type versus CR030 (Δ*cysR*) were found to be differentially expressed anymore, indicating that there is indeed strong indirect regulation caused by the other two regulatory networks. For example, the initially observed decrease in transcript levels of the *ssu*/*seu *genes in the Δ*cysR *mutant is most likely due to a decrease in transcription of the activator gene, *ssuR*, and not due to direct control by CysR, validating the complex experimental approach.

In addition to the McbR-regulated genes with increased transcription, quite surprisingly, one gene of the McbR regulon, *cg2810*, was found to be significantly less transcribed in the mutant with intact CysR. Along with the even stronger repressed genes *cg3138-39*, this observation delivered the first indication that CysR might act not only as a transcriptional activator, but also as a repressor.

To validate these results, the ratios of the mRNA levels of genes of interest that belong to at least one of the regulons of McbR, SsuR, or possibly CysR were determined using real-time RT-PCR (Tab. 1). This approach confirmed the data from the microarray hybridisations, indicating that the CysR regulon consists of at least seven transcription units, by name *cysIXHDNYZ*, *fpr2*, *ssuR*, *cg1514-cg4005*, *cg2810*, *cg3138-39*, and *3372-75*.

### 2.3 *O*-acetyl-L-serine (OAS) and *O*-acetyl-L-homoserine (OAH) are required as effector substances for the binding of the regulator CysR to DNA

**Figure 3:**
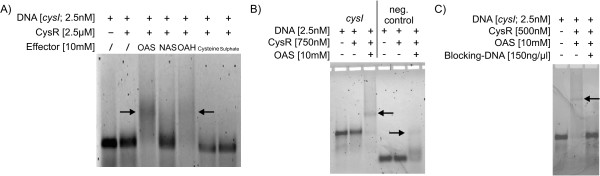
**Electrophoretic mobility shift assays using purified CysR to identify the co-activators of the protein (A) and test for specific binding (B+C)**. CysR was purified using the IMPACT-C system (New England Biolabs) in presence of 0.1% Triton X-100. A Cy3-labelled 523 bp fragment containing the *cysI *- *fpr2 *intergenic region was incubated with a 1,000 times molar excess of the purified protein in presence of different intermediates of sulphur metabolism (OAS, *O*-acetyl-L-serine; NAS, *N*-acetyl-L-serine; OAH, *O*-acetyl-L-homoserine) at 10 mM concentration, demonstrating that CysR can only bind to DNA in presence of either OAS or OAH (A). Tests with OAS-activated CysR and either a negative control (123 bp internal fragment of *cg2118*, B) or herring sperm blocking DNA (C) revealed that activated CysR binds non-specifically *in vitro*.

With the transcriptomic studies showing that the mRNA pools of several transcription units are significantly altered in response to the presence of an intact CysR, the direct interaction of CysR with DNA had to be verified. In addition, possible effectors of CysR and the binding site(s) should be identified. As a first approach to solve these questions, gel retardation experiments were carried out. Therefore, CysR was expressed using the IMPACT system (New England Biolabs; Ipswich, MA) fused with a C-terminal intein/chitin-binding domain. After extensive optimisation of the cultivation to avoid inclusion body formation as well as the cell lysis and elution steps (see *Experimental procedures*), CysR was purified to homogeneity, carrying only one additional glycine residue at the C-terminal end. Interestingly, CysR could only be eluted in presence of low concentrations of detergents like Triton X-100 (data not shown). In EMSA studies, Cy3-labelled PCR products of the genomic DNA regions between *cysI *and *fpr2 *as well as upstream of *ssuR*, *cg3372*, *cg1514*, and *cg2810 *(approx. 550 bp each) were incubated with this purified protein and then separated by agarose gel electrophoresis, but no bandshift was observed (data not shown). Previous studies in *E. coli *had revealed that activation of the pathway for assimilatory sulphate reduction by CysB is dependent on the presence of *N*-acetyl-L-serine (NAS), a derivative of the intermediate OAS [27]. As CysR is thought to be the functional analogue of CysB, we tested binding of CysR to the region upstream of *cysI *in presence of various intermediates in the biosyntheses of L-cysteine and L-methionine (Fig. 3A). This approach revealed that CysR can only bind if either OAS or OAH is added to the assay while addition of L-cysteine, sulphate, and, in contrast to *E. coli*, NAS did not affect binding.

This indicates that CysR can only activate transcription of sulphate assimilation genes if acceptor molecules for the produced sulphide are available.

After establishing *in vitro *binding of CysR to DNA, we proceeded to use this system to identify the CysR binding site(s) in front of the regulated genes. Unfortunately, subsequent tests with OAS-activated CysR led to the finding of an indiscriminate binding of the activated protein to DNA *in vitro*: activated CysR binds as well to Cy3-labelled negative controls (e.g., to an internal fragment of *cg2118 *[28]; Fig. 3B) as to unlabelled blocking DNA (Fig. 3C). Therefore, while useful to identify the presumable co-activators of CysR, for determination of the binding sites the *in vitro *approach had to be abandoned in favour of an *in vivo *test system.

### 2.4 A 10 bp inverted repeat is present in the mapped promoter/operator region of CysR-controlled genes

**Figure 4:**
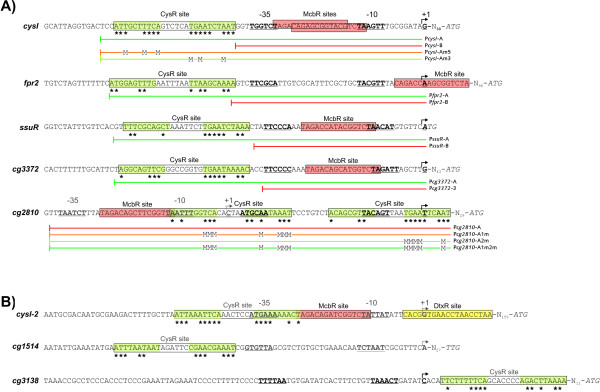
**Mapped (A) and predicted (B) promoter regions of CysR-controlled genes**. Transcription start sites (bold, marked with an arrow and +1) were mapped using the RACE method. The -10 and -35 elements were then inferred either from this transcription start site (bold black) or the location of either McbR [7] or CysR binding sites (grey elements), based on the published consensus sequences determined by Patek *et al*. [29]. The potential CysR binding sites are boxed, the inverted repeat is highlighted in light green with matching bases marked with asterisks. Coloured lines mark the regions cloned into pRIM2 for *in vivo *promoter tests, bases mutated by transition are indicted with M.

In order to identify the CysR sites *in vivo*, as a first step mapping of the transcription start sites was carried out to detect the structural elements of possible promoters and operators. We focused on those genes that are also part of the McbR regulon, as the McbR binding sites are often located between the -35 and -10 regions of the promoter [7], thus facilitating the identification of these elements. As the start sites of transcription for *cysIXHDNYZ *and *fpr2 *had been mapped previously [24], 5'-RACE was used to determine those of the remaining three McbR-regulated transcription units. In all three cases, one single start site could be determined for each transcription unit (Fig. 4A). In the case of *ssuR *and *cg3372*, the McbR binding site is located directly between the -35 and -10 elements derived from the *σ*^A ^consensus sequence [29]. But in the case of *cg2810*, the McbR binding site is located 14 bp upstream of the predicted -35 region. Closer inspection of the region around the McbR binding site in this case revealed a second, hypothetical promoter that matches closely to the *σ*^A ^consensus binding motif.

**Figure 5:**
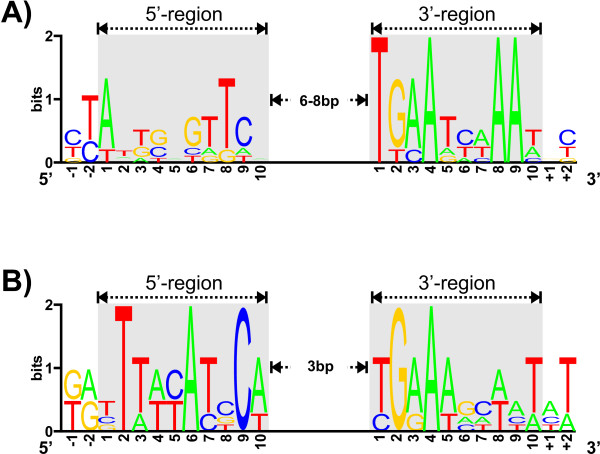
**Sequence logos of (A) the predicted CysR binding site (n = 6) and (B) the extended SsuR binding site (n = 4)**. (A) The conserved TGAAtnnAAt motif of the 3'-region (gray box) has a high information content (total height of the characters), which can be assumed to be equivalent to high biological importance. In contrast, the bases flanking the motif are more or less random, resulting in a very low information content. The 5'-box is only partially conserved but still discernible from the adjacent sequences. (B) The 3'-region of the SsuR binding site (consensus TGaA [at]nnaaT) is very similar to that of recognised by CysR. Noticeable differences include the much higher degree of conservation in the 5'region as well as in the spacing of the regions of 3 bp in every known instance.

With the transcription start sites mapped, the regions upstream of the -35 elements of activated promoters (P_*cysIXHDNYZ*_, P_*fpr*2_, P_*ssuR*_, and P_*cg*3372_) were searched for the presence of conserved DNA motifs. In all four cases, two 10 bp motifs that form an inverted repeat and are separated by 6 – 8 bp were found 3 bp upstream of the -35 region (Fig. 4A). As *cg2810 *is thought to be repressed by CysR, a search for motifs similar to this inverted repeat inside and downstream of the *cg2810 *promoter regions was performed, identifying two candidate motifs that overlap with the identified as well as the hypothetical promoter (Fig. 4A). Based on these six instances, a multiple alignment of the six potential binding sites was created and visualised as a sequence logo (Fig. 5A; [30]). This approach indicates that the inverted repeat consists of a highly conserved 3'-region while the 5'-region appears to be less conserved, resulting lower information content. Interestingly, this motif is similar to one present in the binding sites we identified for the paralogous transcriptional activator SsuR [15]. A multiple alignment of the SsuR binding sites including two additional base pairs downstream of the binding site revealed regions which closely match the inverted repeat that represents the hypothetical CysR binding site (Fig. 5B). Marked differences include the degree of conservation of several bases in both regions, the higher degree of conservation in the 5'-region of the SsuR binding motif and especially the much smaller distance between the two inverted repeats of only 3 bp.

### 2.5 CysR-dependent regulation requires the presence of the identified 10 bp inverted repeat *in vivo*

To verify these inverted repeats as binding sites for CysR, several promoter test plasmids based on the promoter-probe vector pRIM2 [31] were constructed. This vector allows to determine promoter activity by transcription of a promoterless *cat *gene encoding chloramphenicol acetyltransferase that can be measured either by real-time RT-PCR on the level of the mRNA or by Cat-ELISA (enzyme linked immuno sorbent assay) on the protein level. In the current case, two plasmids containing either the core promoter without the inverted repeat or the promoter/operator region including the potential binding site (Fig. 4A; indicated by coloured bars) were assembled for each of the four activated transcription units.

In the case of the repressed gene, *cg2810*, a different approach was applied, as the potential CysR binding sites overlap with elements of both promoters. Therefore, the binding sites could not be completely removed but were mutated by several transitions at positions outside of the -35 and -10 regions in either one or both of the two possible CysR binding sites (Fig. 4A; coloured bars, mutated bases are marked by M). Finally, to collate the relative importance of the two 10 bp motifs, two mutated versions of the *cysI *promoter/operator region were constructed, each containing three transitions in either the 5'- or the 3'-motif at conserved positions (Fig. 5A).

**Figure 6:**
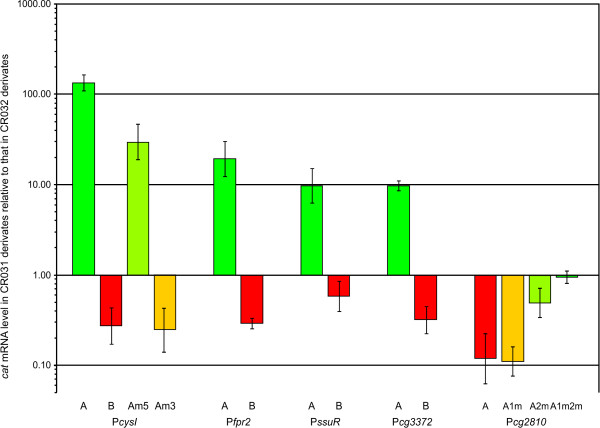
**Relative *cat *mRNA levels in pRIM2::promoter-carrying *C. glutamicum *CR031 strains compared to the corresponding *C. glutamicum *CR032 strains**. Strains CR031-035i to CR031-048i and CR032-035i to CR032-048i carrying different promoter fragments cloned in pRIM2 in front of a promoterless *cat *gene were grown in MMS minimal medium with limiting amounts of sulphur. Details of the A, B, Am5 or Am3 form of the cloned promoter fragments are shown in Fig. 4. After 30 min sulphur starvation, cells were harvested, total RNA was isolated, and the relative *cat *mRNA amounts were determined using real-time RT-PCR. The values are the means of at least two independent biological samples with two technical replicates each.

Each of the resulting 14 plasmids was transferred into the *C. glutamicum *strains CR031 (constitutively expressing CysR) and CR032 (*cysR*^ΔHTH ^mutant) by electroporation, yielding strains CR031-035i to CR031-048i and CR032-035i to CR032-048i, respectively. Growth of the resulting 28 strains, RNA preparation and relative quantification of mRNA levels using real-time RT-PCR were performed as detailed in *Experimental procedures*. By comparing the amount of *cat *mRNA in strain CR031 (carrying an intact CysR) to that in strain CR032 (CysR inactive due to missing HTH domain), the effect of CysR on the different promoter constructs can be determined. Using this approach, it was demonstrated that the predicted binding sites are indeed involved in CysR-mediated control:

Only if the cloned fragment contains also the predicted binding site (Fig. 6, promoters labelled with A), the relative *cat *mRNA amount is significantly increased when *cat *is under control of the promoters of *cysI*, *fpr2*, *ssuR*, and *cg3372*.

Additional evidence for the importance of the inverted repeat was added by the two mutated forms of the *cysI *promoter: Three transitions in conserved bases in the 5'-box reduce CysR-mediated induction of the promoter to 22% of the induction level observed with the intact operator region (Fig. 6, P*cysI *-A and -Am5), while transitions in three conserved bases of the 3'-box reduce the relative *cat *level to that observed with the core promoter (Fig. 6, P*cysI *-B and -Am3).

This data is further supported by the results obtained for the negatively regulated promoter of *cg2810*: The cloned promoter with both potential binding sites intact is significantly repressed in presence of an intact CysR (Fig. 6, P*cg2810*-A). This repression does not decrease if the distal of the two binding sites is mutated (as the proximal site can still block transcription), but mutation of the proximal site results in a significant de-repression and loss of both sites completely abolishes transcriptional control by CysR. All in all, the observed changes in the relative *cat *levels strongly support the hypothesis that the inverted repeat is involved in CysR-mediated regulation, especially as the relative changes in the *cat *mRNA levels correspond to the observed changes of the native transcripts (Table 1). A surprising discovery is the observation that the relative *cat *mRNA level actually decreases significantly in strains expressing an intact CysR if the *cat *gene is transcribed only via the "core" promoter of one of the activated genes (Fig. 6, promoters labelled with B). The cause of this effect was not identified so far but might be linked to RNA polymerase availability [32].

**Table 1:** Relative expression levels of genes belonging to the regulons of McbR, SsuR, and/or CysR measured by real-time RT-PCR

CDS	Name	Regulon^a^	Function	mRNA ratio WT/CR030	comparing^b ^CR031/CR032
*Cg0012*	*ssuR*	McbR, CysR	Transcriptional activator of sulphonate(ester) utilisation	32.9	12.8
*Cg0156*	*cysR*	McbR	Transcriptional activator of assimilatory sulphate reduction	0.61	0.76
*Cg0755*	*metY*	McbR	*O*-acetylhomoserine sulfhydrylase	1.5	1.0
*Cg1514*		CysR*	Secreted protein of unknown function	137.9	158.1
*Cg1376*	*ssuD1*	McbR, SsuR	FMNH_2_-dependent aliphatic sulphonate monooxygenase	186.8	1.3
*Cg2810*		McbR, CysR	Putative secondary H^+^/Na^+ ^symporter	0.39	0.14
*Cg3118*	*cysI*	McbR, CysR	Ferredoxin-sulphite reductase	104.7	93.4
*Cg3119*	*fpr2*	McbR, CysR	Ferredoxin-NADP^+ ^reductase	21.6	8.8
*Cg3138*		CysR*	Putative integral membrane protein	0.0078	0.018
*Cg3253*	*mcbR*	McbR	Transcriptional repressor of sulphur metabolism	0.97	0.88
*Cg3372*		McbR, CysR	Conserved protein of unknown function	99.0	22.9

^a ^According to the studies of [7,15], and this study.

^b ^Total RNA was isolated from *C. glutamicum *cultures grown in minimal medium with 2 mM L-cysteine as sole sulphur source to o.D._600 _10 and then subjected to sulphur starvation for 30 min. Relative expression levels were measured for two independent replicates with at least three technical replicates each.

* predicted from microarray data and motif search.

### 2.6 The proposed regulon of the dual regulator CysR consists of seven transcription units

To identify additional putative binding sites, a search for the motif in the upstream regions of genes with significantly changed transcription level in the microarray studies was carried out, using the program Fuzznuc [33]. Only three instances of a possible binding site were found in these searches, all of which are located in the upstream regions of the most strongly regulated genes, by name *cg1514*, *cg3138*, and *cysI *. In case of the strongly regulated transcription units *cg1514-cg4005 *and *cg3138-39*, the degree of conservation of the predicted site is rather low (Fig. 4B). In both cases, a putative promoter is located either downstream of the putative binding site (in case of *cg1514*) or upstream of the binding site (for *cg3139*), which corresponds to the observed induction respectively repression in the microarray data.

Interestingly, a second, well conserved binding site far upstream of *cysI *was found. This site overlaps with a putative promoter and that would be repressed in presence of CysR as well as by McbR and DtxR [34]. The function of this second promoter remains to be determined, but it might be used to provide a basal transcription in absence of CysR: as it matches more closely to the *σ*^70 ^binding site [29] than the proximal, CysR-activated promoter, it should allow transcription even if CysR is missing.

**Figure 7:**
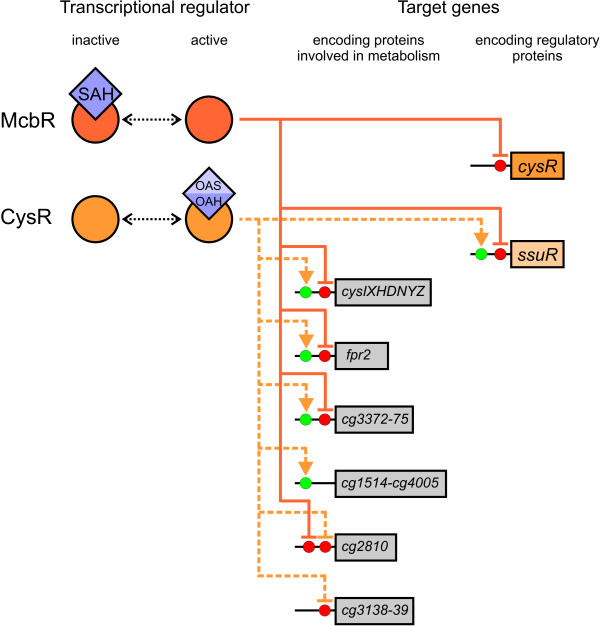
**Schematic model of the transcriptional regulation of sulphate reduction in *C. glutamicum***. The transcriptional repressor McbR acts as a top level regulator, controlling the transcription of its regulon in dependence of the effector *S*-adenosyl-L-homocysteine (SAH). Absence of SAH results in repression of gene transcription by active McbR while presence of SAH leads to inactivation of McbR. This allows at least basic transcription of all McbR-controlled genes, among them those encoding the regulatory proteins CysR and SsuR as well as those involved in assimilatory sulphate reduction (*fpr2 cysIXHDNYZ*). The second level of regulation is monitored by CysR that controls several genes in response to the availability of either *O*-acetyl-L-serine (OAS) or *O*-acetyl-L-homoserine (OAH), activating at least five transcription units while repressing two others. Among the controlled genes are those needed for assimilatory sulphate reduction, the one encoding the transcriptional regulator SsuR, and several genes of unknown function. Transcriptional regulators are represented by circles and effector metabolites are given as diamonds. Genes are displayed as boxes with transcriptional activation marked with arrows and repression marked with dashes.

In total, the gathered data indicates that at least seven transcription units are putatively controlled by the transcriptional regulator CysR, five of which are activated while two are repressed in presence of activated CysR (Fig. 7).

## 3 Discussion

### 3.1 The CysR protein is involved in the activation of the pathway for assimilatory sulphate reduction and possibly related genes in *C. glutamicum*

In this study the *C. glutamicum *gene *cysR *(*cg0156*) was analysed, a gene which drew attention due to being part of the regulon of the global regulator of sulphur metabolism, McbR [7]. Transcription of the likewise McbR-repressed gene cluster *fpr2 cysIXHDNYZ*, which was found to be necessary for assimilatory sulphur reduction in *C. glutamicum *[24], was found to be dependent on the presence of a functional CysR protein. This, the gathered *in vivo *data, and the similarity to the ROK-type transcriptional activator SsuR [15] strongly indicates that CysR acts as transcriptional activator of these two transcription units, albeit a specific interaction of CysR with the predicted binding motif could not be shown *in vitro*. In addition, the obtained data indicates that CysR is involved in the transcriptional regulation of at least five other transcription units, activating three while repressing two others, depending on the location of the CysR binding site relative to the promoter. This puts CysR in the class of dual transcriptional regulators that can act as repressors and activators, depending on the localisation of the binding site, a class of regulators found more and more to be common in *C. glutamicum *[35].

Concerning the uncharacterised members of the CysR regulon, only general assumptions can be made based on the annotated functions. In case of the strongly induced genes *cg1514 *and *cg4005*, the encoded proteins are thought to be secreted and Cg1514 was indeed found in the extracellular proteome [36]. As the known genes of the regulon are involved in providing the cell with reduced sulphur, it stands to reason that Cg1514 and Cg4005 might be involved in some sort of sulphur scavenging.

For the repressed gene *cg2810*, a possible function as transporter can be proposed. According to UNIPROT and PFAM it belongs to the sodium/proton-dicarboxylate symporter family which is also involved in the uptake of amino acids [37]. Based on the observation that Cg2810 is repressed by CysR, a function as the low affinity transporter for cysteine and/or cystine seems possible. This transporter would not be necessary if the cells are starved for sulphur as not enough extracellular cyst(e)ine would be available under those conditions.

For the two remaining transcription units, *cg3372-75 *and *cg3138-39*, no function in sulphur metabolism can be inferred from either the annotation or bioinformatic analyses. Still, for all these "novel" genes, a detailed functional analysis might provide interesting new insights into sulphur metabolism, especially of the genes *cg3372-75 *and *cg2810*. As these genes are part of not only the CysR but also of the McbR regulon, it can be assumed that they play a major role in sulphur metabolism of *C. glutamicum*.

### 3.2 The regulator CysR is a member of the ROK protein family

The CysR protein from *C. glutamicum *displays similarity to transcriptional regulators of the ROK family [14], although it is at best a distant member. The distance on the sequence level is accompanied with a switch of effectors: ROK-type repressors usually react to sugar intermediates [38,39] while CysR is controlled by *O-*acetylated amino acids. Syntenous orthologues of CysR are present on the genomes of *Corynebacterium efficiens *[40] and *Corynebacterium diphtheriae *[41] while SsuR might be considered a paralogue (which reacts to inorganic sulphate).

While similarity on the protein level is rather weak, there is a strong correlation between XylR (repressors of xylose metabolism in the *Firmicutes *[38]), Mlc and NagC (which control the phosphotransferase system in *E. coli *[42]), SsuR, and CysR on the level of the binding motif. For Mlc and NagC, it was shown that they usually bind to inverted repeats consisting of a T-rich 5'-region and a correspondingly A-rich 3'-region separated by a variable length GC-rich core [42]. In case of CysR as well as for SsuR and XylR binding sites, the A- and T-rich regions seem to be conserved while the elevated GC-content of the core is not present in several instances, indicating that the inverted repeat is more important than the separating spacer. But this spacer might play a role in the discrimination between CysR and SsuR binding sites as it is the only consistent difference between the two motifs. The otherwise high degree of similarity of the binding motifs as well as the amino acid sequence similarity back the theory that these two regulators are indeed paralogues, adding a dual regulator to the heterogeneous ROK protein family.

### 3.3 The *cysR *gene is transcribed as leaderless transcript

A interesting finding was that the mapped transcriptional start sites of *cysR *as well as of *ssuR *are identical with the translation start points of the encoded proteins, adding them to the growing number of leaderless transcripts of *C. glutamicum*. The transcripts of several *C. glutamicum *genes which encode proteins involved in amino acid metabolism were shown to belong to this transcript type [29] as well as the genes necessary for sulphonate (but not sulphonate ester) utilisation [15]. The translation efficiency of leaderless transcripts depends on several factors, especially the ratio of the initiation factors IF2 and IF3 [43]. This would couple efficient translation of *cysR *and *ssuR *to the growth rate as the IF2:IF3 ratio is thought to be high during fast cell growth [43]. As it has been speculated that McbR inactivation, and thereby *cysR *and *ssuR *transcription, is also linked to the growth rate via the effector of McbR, SAH [2], it stands to reason that CysR and thereby SsuR as well as their respective regulons can be expressed efficiently only in fast growing cells.

### 3.4 The presence of sulphide acceptor molecules is necessary for binding of CysR to DNA

The effector studies clearly demonstrated that CysR binds to DNA only in the presence of effector metabolites *in vitro*. OAS and OAH were demonstrated to enable binding of CysR to DNA, albeit in a non-specific manner *in vitro*. Both OAS and OAH are direct acceptor molecules for sulphide, the product of the CysR-activated pathway for assimilatory sulphate reduction. Thus, production of highly toxic sulphide is initiated only if it can be directly converted to less toxic compounds, by name L-cysteine and L-homocysteine, protecting the cells from sulphide accumulation. This mechanism is well known from, e.g., *E. coli*: transcription of the genes encoding the sulphate reduction pathway is dependent on the LysR-type regulator CysB [44] which is in turn modulated by the OAS-derived metabolite *N*-acetyl-L-serine (NAS). While CysB can bind to DNA in absence of NAS, activation of transcription is dependent on that metabolite [45]. Due to the presence of two acceptor substances for sulphide in *C. glutamicum*, the observed direct sensing of these metabolites is a logical extension of the regulatory network.

### 3.5 The deduced regulatory model of CysR in *C. glutamicum*

The collected data of the present and previous studies [6,7,15,16,24] allows to build a model of the regulation of sulphur metabolism in *C. glutamicum *(Fig. 7). Almost all genes known to be involved in sulphur metabolism are negatively controlled by the global transcriptional repressor McbR. McbR is in turn negatively controlled by SAH which is thought to link sulphur metabolism to cell growth (reviewed in [2]). With an increasing SAH pool, transcription of the genes under McbR control is derepressed, resulting in the increased expression of the genes needed to synthesise the sulphur-containing amino acids. In addition, the two ROK-type regulators CysR and SsuR are expressed, providing the basis for the subsequent regulatory cascade. The prerequisite to trigger the next step of the cascade is the accumulation of either OAS or OAH which are needed for CysR-mediated gene regulation. Both act as acceptor molecules for sulphide leading to L-cysteine and L-homocysteine in sulphydrylase-catalysed reactions. Therefore, the CysR-controlled pathway for assimilatory sulphate reduction is only activated if the produced sulphide can be directly converted to less toxic compounds. In turn, the syntheses of both OAS and OAH are strictly regulated. In case of OAH, the regulation occurs on the level of transcription by McbR-mediated control of homoserine *O*-acetyltransferase (MetX [7,46]). On the other hand, OAS synthesis is controlled by L-cysteine-mediated feedback-inhibition of serine *O*-acetyltransferase (CysE [47]). In addition to assimilatory sulphate reduction, CysR might also be involved in the activation of sulphur scavenging and repression of a possible low-affinity amino acid transporter.

Finally, CysR-mediated activation of *ssuR *transcription is necessary to provide for enough SsuR activator to trigger the bottommost level of regulation. If the available sulphate becomes limiting, SsuR becomes active and induces expression of the genes involved in uptake and utilisation of sulphonates and their esters, providing *C. glutamicum *with an alternative supply of sulphur, usually abundant in soils [15]. Interestingly, the regulatory network of sulphur metabolism in *C. glutamicum *shares features of the networks of those described for *E. coli *and *Bacillus subtilis*: *E. coli *lacks a global regulation of sulphur metabolism realised in *B. subtilis *with the S-box riboswitch [9] and in *C. glutamicum *with the McbR regulon. On the other hand, the current model of a staggered response of the corynebacterial ROK-type activators CysR and SsuR is strongly reminiscent of the two unrelated LysR-type regulators CysB and Cbl found in *E. coli *[48,49] while the McbR/CysR regulon shares a similar topology like the MetJ/MetR regulon in *E. coli *[50].

These similarities in network topology in remote bacterial phyla indicate a strong selective pressure to evolve such a topology. This is most evident for the analogous regulator pair CysB/Cbl from *E. coli *and CysR/SsuR from *C. glutamicum*: It stands to argue that the activators of sulphonate utilisation, Cbl respectively SsuR, are each a result of a gene duplication event. The resulting paralogues then took control of the genes needed for sulphonate utilisation. This hypothesis is backed by the similarity on both the protein level and the similarity of the binding sites. In *E. coli*, this results in a still present direct regulation of Cbl-controlled genes by CysB [49,51] while control of the *ssu *and *seu *by CysR has become indirect in *C. glutamicum*.

## Conclusion

With the identification of CysR, the "missing link" in the regulation of sulphur metabolism in *C. glutamicum *is now known, expanding our understanding of the complex regulation of this metabolic module and delivering new, interesting targets for future functional studies like the CDS *cg1514*, *cg2810*, *cg3138*, and *cg3372*.

Concerning the regulation of sulphur metabolism in general, the extension of the characterised members ROK family is interesting in itself due to the unusual effectors (for the ROK family). Still, the convergent evolution of the regulatory networks of the completely unrelated transcriptional regulators CysR/SsuR (ROK-type) in *C. glutamicum *and, CysB/Cbl (LysR-type) in *E. coli *is the most striking finding. Besides the similarity of the regulons, both regulators recognise similar effector molecules and share the same network topology which seems to be due to independent gene duplication and specialisation events of the respective ancestral regulators. This indicates that there is a strong selective pressure to tightly regulate and balance the metabolism of the essential (but often toxic) sulphur compounds.

## 4 Methods

### 4.1 Bacterial strains, plasmids and culture media

The bacterial strains and plasmids used in this study are listed in Table 2. *E. coli *strains carrying plasmids were routinely grown on solid Antibiotic Medium No. 3 (PA) (Oxoid, Wesel, Germany) at 37°C. *C. glutamicum *strains were grown on solid brain-heart broth (BH) (VWR International, Darmstadt, Germany) at 30°C. For growth tests in liquid medium, MMES minimal medium [24] was used with addition of different sulfur sources at 100 *μ*M concentration. Cultivations for RNA harvesting were performed in MMS minimal medium [24] with addition of L-cysteine, corn steep liquor (CSL) and yeast extract (YE). CSL/YE were used as a 20%/20% stock solution derived by diluting CSL (Sigma-Aldrich, Taufkirchen, Germany), adding YE, removing solids by filtration and centrifugation and final sterilisation by filtration.

**Table 2:** Bacterial strains and plasmids

Name	Relevant genotype/information^a^	Source/reference
*E. coli *DH5αMCR	F^- ^*endA1 supE44 mcrA thi-1 hsdR17λ*^- ^*recA1 relA1 *Δ(*lacZYA-argF*) *U169 *(Φ80d*lacZ*Δ*M15*) *gyrA96 deoR *Δ(*mrr-hsdRMS-mcrBC*)	[58]
ER2566	F^- ^*λ*^- ^*fhuA2 [lon] ompT lacZ::T7 gene1 gal sulA11 *Δ*(mcrC-mrr)114::IS10 R(mcr-73::miniTn10-TetS)2 R(zgb-210::Tn10)(TetS) endA1 [dcm]*	New England Biolabs
		
*C. glutamicum*		
ATCC 13032	Wild type, Nx^R^	ATCC^b^
CR030	Δ*cysR*	this study
CR031	*mcbR*^ΔHTH ^*ssuR*^ΔHTH ^*cysR*^const^	this study
CR032	*mcbR*^ΔHTH ^*ssuR*^ΔHTH ^*cysR*^ΔHTH^	this study
CR031-035i	CR031 with integrated pCR035i, Km^R^	this study
CR031-036i	CR031 with integrated pCR036i, Km^R^	this study
CR031-037i	CR031 with integrated pCR037i, Km^R^	this study
CR031-038i	CR031 with integrated pCR038i, Km^R^	this study
CR031-039i	CR031 with integrated pCR039i, Km^R^	this study
CR031-040i	CR031 with integrated pCR040i, Km^R^	this study
CR031-041i	CR031 with integrated pCR041i, Km^R^	this study
CR031-042i	CR031 with integrated pCR042i, Km^R^	this study
CR031-043i	CR031 with integrated pCR043i, Km^R^	this study
CR031-044i	CR031 with integrated pCR044i, Km^R^	this study
CR031-045i	CR031 with integrated pCR045i, Km^R^	this study
CR031-046i	CR031 with integrated pCR046i, Km^R^	this study
CR031-047i	CR031 with integrated pCR047i, Km^R^	this study
CR031-048i	CR031 with integrated pCR048i, Km^R^	this study
CR032-035i	CR032 with integrated pCR035i, Km^R^	this study
CR032-036i	CR032 with integrated pCR036i, Km^R^	this study
CR032-037i	CR032 with integrated pCR037i, Km^R^	this study
CR032-038i	CR032 with integrated pCR038i, Km^R^	this study
CR032-039i	CR032 with integrated pCR039i, Km^R^	this study
CR032-040i	CR032 with integrated pCR040i, Km^R^	this study
CR032-041i	CR032 with integrated pCR041i, Km^R^	this study
CR032-042i	CR032 with integrated pCR042i, Km^R^	this study
CR032-043i	CR032 with integrated pCR043i, Km^R^	this study
CR032-044i	CR032 with integrated pCR044i, Km^R^	this study
CR032-045i	CR032 with integrated pCR045i, Km^R^	this study
CR032-046i	CR032 with integrated pCR046i, Km^R^	this study
CR032-047i	CR032 with integrated pCR047i, Km^R^	this study
CR032-048i	CR032 with integrated pCR048i, Km^R^	this study
		
Plasmids		
pK18*mobsacB*	*sacB*, *lacZa*, Km^R^, mcs	[56]
pRIM2	promoterless *cat*, Km^R^, d*ppc*	[31]
pTYB1	Ap^R^, carrying an intein-coupled chitin binding domain	New England Biolabs
pCR030d	pK18*mobsacB *carrying *cysR *del^c^	this study
pCR031d	pK18*mobsacB *carrying *ssuR *HTHdel	this study
pCR032d	pK18*mobsacB *carrying *cysR *HTHdel	this study
pCR033d	pK18*mobsacB *carrying *mcbR *HTHdel	this study
pCR034d	pK18*mobsacB *carrying *cysR *const^d^	this study
pCR035i	pRIM2 carrying P_*cysI*-A_	this study
pCR036i	pRIM2 carrying P_*cysI*-B_	this study
pCR037i	pRIM2 carrying P_*cysI*-Am5_	this study
pCR038i	pRIM2 carrying P_*cysI*-Am3_	this study
pCR039i	pRIM2 carrying P_*fpr*2-A_	this study
pCR040i	pRIM2 carrying P_*fpr*2-B_	this study
pCR041i	pRIM2 carrying P_*ssuR*-A_	this study
pCR042i	pRIM2 carrying P_*ssuR*-B_	this study
pCR043i	pRIM2 carrying P_*cg*3372-A_	this study
pCR044i	pRIM2 carrying P_*cg*3372-B_	this study
pCR045i	pRIM2 carrying P_*cg*2810-A_	this study
pCR046i	pRIM2 carrying P_*cg*2810-A1m_	this study
pCR047i	pRIM2 carrying P_*cg*2810-A2m_	this study
pCR048i	pRIM2 carrying P_*cg*2810-A1m2m_	this study
pCR050	pTYB1 carrying a *cysR*-intein fusion with a C-terminal glycine	this study

^a ^r superscript indicates resistance. Nx, Nalidixic acid; Km, Kanamycin

^b ^ATCC; American Type Culture Collection, Rockville, MD

^c ^the postfix *del *indicates inserts used for targeted gene deletion.

^d ^the postfix *const *indicates inserts used for promoter replacement

Antibiotics used for selection of plasmids and strains were nalidixic acid (50 *μ*g/ml for corynebacteria) and kanamycin (50 *μ*g/ml for *E. coli*, 25 *μ*g/ml for corynebacteria).

### 4.2 DNA isolation, transfer and manipulation

Standard procedures were employed for molecular cloning and transformation of *E. coli *DH5*a*, as well as for electrophoresis [52]. Transformation of *C. glutamicum *was performed by electroporation using the methods published previously [53].

### 4.3 Polymerase chain reaction experiments

PCR experiments were carried out with either BioTaq *Taq *DNA polymerase (Bioline, Luckenwalde, Germany) for control reactions or with Phusion high-fidelity DNA polymerase (New England Biolabs, Frankfurt a. Main, Germany) for DNA fragments to be used for subsequent cloning experiments. As templates, chromosomal *C. glutamicum *DNA, isolated according to [54], and pK18*mobsacB *(for amplification of the *neo *promoter) were used. Oligonucleotides used as primers were purchased from Operon Biotechnologies (Cologne, Germany). All PCR setups were done according to the manufacturers protocols.

### 4.4 Construction of plasmids

Plasmids pCR030d to pCR034d were constructed using the gene splicing by overlap extension (gene-SOEing) method described in [55] with modifications as described previously [24]. In case of pCR034d, the native promoter of *cysR *was replaced with the *neo *promoter of pK18*mobsacB*. Plasmids pCR035i to pCR048i were constructed by using *Spe*I and *Bgl*II restriction sites added by the PCR primers used to amplify the respective promoter fragments. After restriction cleavage the inserts were ligated into *Xba*I – *Bam*HI digested pRIM2.

Ligation mixtures were used for transformation of *E. coli *DH5*a*MCR, the transformants were selected on PA plates containing 50 *μ*g/ml kanamycin and, if appropriate, 40 mg/l X-Gal.

### 4.5 Site-specific gene deletion/promoter replacement

Site-specific gene deletion was performed using the non-replicable integration vector pK18*mobsacB *which allows for marker-free deletion of the target gene [56]. The plasmids pCR030d to pCR034d were transferred into *C. glutamicum *strains by electroporation [53]. Tests for first and second cross-over were performed as described previously [46].

### 4.6 Real-time monitoring of cell growth using nephelometry

Nephelometry was performed as described in [24], with sulphur sources used at concentrations of 100 *μ*M each. Per strain and condition, at least 3 biological replicates (plates) were measured, with 6 technical replicates (wells) per plate.

### 4.7 RNA preparation and DNA microarray hybridisation

Bacterial cell cultures were inoculated in MMS with addition of 0.01% corn steep liquor and 0.01% yeast extract, containing 2 mM L-cysteine as sulphur source. The cultures were grown to the early logarithmic phase (o.D.600 8 – 12) in a Innova 4430 orbital shaker (New Brunswick, NJ) at 300 rpm, and 30°C using 10 ml medium in a 100 ml Erlenmeyer flask.

For each experiment, 10^10 ^cells were pelletised by centrifugation, washed once with MMS without added sulphur source (preheated to 30°C), resuspended in 10 ml preheated MMS without addition of sulphur, and incubated for an additional 30 min.

For RNA isolation, about 10^9 ^cells per culture were harvested by 15 sec centrifugation at 16,000 g, followed by immediate removal of the supernatant and freezing of the pellet in liquid nitrogen. Preparation of total RNA from *C. glutamicum *cells, cDNA synthesis, and array hybridisation were performed as described in [25], using 70 mer oligo microarrays instead of dsDNA microarrays. Evaluation of the hybridisation experiment was done as described in [7], using a m-value cut-off of ± 1, which corresponds to expression changes equal or greater than twofold. The scanned arrays were analysed with IMAGENE v6.0 (BioDiscovery Inc.; El Segundo, CA) and statistical analyses were carried out using the Emma2 software [57].

### 4.8 Purification of the CysR protein

To purify native CysR protein, the coding region was cloned into the pTYB1 IMPACT vector (New England Biolabs, Frankfurt a. Main, Germany). As the C-terminal proline of CysR would block intein-mediated cleavage, an additional C-terminal glycine was added via the primer. The resulting plasmid was transferred into the expression host strain *E. coli *ER2566 via electroporation. For protein purification, 4 aliquots of 250 ml Luria-Bertani Broth (LB) medium (Oxoid, Wesel, Germany) containing 100 *μ*g/ml ampicillin were inoculated with 2.5 ml freshly grown overnight culture each (also in LB with addition of 100 *μ*g/ml ampicillin) and transferred to 1,000 ml Erlenmeyer flasks. The cultures were grown in a Thermoshake orbital shaker (Gerhardt Analytical Systems, Bonn, Germany) at 150 rpm and 37°C to an o.D.600 of 0.5 – 0.6. To these cultures IPTG was added to a final concentration of 0.5 mM and the cultivation temperature was reduced to 15°C. After growth overnight, cells were harvested by centrifugation (15 min with 6,000 g at 4°C).

The pellets were collected in 30 ml pre-chilled lysis buffer (20 mM sodium phosphate and 500 mM NaCl, pH 8.0, with addition of 20 *μ*M PMSF, 1 mM TCEP, and 0.1% Triton X-100) and the cells were lysed using a FRENCH Press with a pre-cooled 40 K Cell (Thermo Electron, Oberhausen, Germany) in two or three passes with a pressure of 1,200 psi. After removal of cell debris by centrifugation (30 min with 6,000 g at 4°C), all further steps were performed according to the IMPACT-CN protocol (New England Biolabs, Frankfurt a. Main, Germany) with the following changes: For washing, phosphate buffer with 1,000 mM NaCl was used to remove all non-specifically bound proteins. Cleavage was performed at 4°C for 16 h. For the elution step, phosphate buffer containing 500 mM NaCl and 0.1% Triton was used, with ≈ 2 bed volumes elution buffer.

The resulting protein solution was concentrated using an Amicon Ultra-4 column (Macherey & Nagel, Düren, Germany) with an exclusion size of 30 kDa and washed thrice with 10 volumes phosphate buffer with 50 mM NaCl to remove excess salt. The purity of the obtained protein was controlled by SDS-PAGE on a 12.5% gel and the identity of the protein was checked by tryptic digestion and MALDI-TOF analysis. Finally, the protein solution was diluted to 5 *μ*M CysR protein, shock-frozen in liquid nitrogen and stored at -80°C.

### 4.9 Electrophoretic mobility shift assays

EMSA studies were performed using Cy3-labelled PCR products that were amplified with appropriate Cy3-labelled 20 mer oligonucleotides and purified with the NucleoSpin Extract II Kit (Macherey & Nagel, Düren, Germany).

For EMSA studies, up to 50 pmol purified CysR protein were added to reaction buffer (1 mM MgCl_2_, 0.5 mM EDTA, 100 mM NaCl, 10 mM Tris, 20% glycerin; pH 7.5) to get a final volume of 19 *μ*l. If additional reagents, e.g. effectors or blocking DNA, were added, the amount of reaction buffer was adjusted accordingly and the assay was incubated for 10 min at room temperature before addition of labelled DNA. Subsequently, 1 *μ*l of a 50 nM solution of purified, Cy3-labelled PCR product was added to the mixture and the assay was incubated for (additional) 10 min at room temperature. The reaction mixture was separated on a 2% agarose gel prepared in gel buffer (20 mM Na_2_HPO_4_; pH 7.0) with a voltage of 14 Vcm^-1 ^applied for 30 min. For Cy3 detection, the gel was scanned with the Typhoon 8600 Variable Mode Imager (Amersham Biosciences Europe, Freiburg, Germany).

### 4.10 Determination of transcriptional starts with the RACE method

Total RNA was isolated from cultures of CR031 and CR032 grown in MMS medium and subjected to sulphur starvation as described below. Primers binding approximately 150 bp and 10 bp downstream of the annotated translational starts of the *cysR*, *ssuR*, *cg2810*, and *cg3372 *genes along with 1.5 *μ*g of total RNA were used for cDNA synthesis. The cDNA was then modified and amplified using the 5'/3' RACE kit (Roche Diagnostics, Mannheim, Germany) according to the supplier's protocol. The obtained PCR products were cloned into the pCR2.1-TOPO vector (Invitrogen, Karlsruhe, Germany) and transferred into *E. coli *DH10B cells [58]. At least four different clones per gene were selected for plasmid preparation and DNA sequencing (IIT Biotech, Bielefeld, Germany).

### 4.11 Relative mRNA quantification using real-time RT-PCR

Growth and harvesting of bacterial cells for total RNA extraction as well as RNA purification were performed as described above. Primers for real-time RT-PCR were constructed to amplify intergenic regions of about 150 bp length of the genes to be analysed. The primers were designed using the Primer Designer 4.2 software (Sci Ed Software, Durham, NC) and were purchased from Operon Biotechnologies (Cologne, Germany).

All real-time RT-PCR experiments were performed using a LightCycler (Roche, Mannheim, Germany) with the Quantace SensiMix One-Step Kit (Quantace, Berlin, Germany). PCR mixes were set up and PCR reactions were performed as described in [16]. All measurements were performed for two biological replicates per condition tested and with two technical replicates per biological replicate. The amounts of the mRNAs of the genes of the cluster were normalised on total RNA (300 ng) and the relative change in transcription rate was determined as 2^-ΔCP ^with ΔCP equal to the difference of the measured crossing points for the test and the control condition.

### 4.12 GenBank/TrEMBL accession numbers

The nucleotide sequences of *cg0156 *from *C. glutamicum *can be found via the genome entry (accession number BX927148). The amino acid sequence of the corresponding protein is available under the accession number CAF18689.

### 4.13 Bioinformatic analysis

Sequence similarity-based searches with nucleotide and protein sequences were performed using BLAST, the Basic Local Alignment Search Tool [59] against the UNIPROT database [60]. Searches using profile Hidden Markov Models (HMMs) from the Pfam database [19] were done using the HMMER software package.

## Authors' contributions

CR wrote the manuscript, performed the bioinformatic analyses and carried out the mutational and transcriptional studies. JM aided the transcriptional studies, purified CysR and performed the electrophoretic mobility shift assays. DJK aided the mutational analysis and performed the growth tests. AA performed the mapping of transcriptional start points. AP aided in coordination and conceived of the design of tables and figures. JK conceived and coordinated this study. All authors read and approved of the final manuscript.
